# Compressive Channel Estimation Based on the Deep Denoising Network in an IRS-Enhanced Massive MIMO System

**DOI:** 10.1155/2022/8234709

**Published:** 2022-09-13

**Authors:** Yong Chen, Fengyuan Jiang

**Affiliations:** School of Electronic and Information Engineering, Lanzhou Jiaotong University, Lanzhou 730070, China

## Abstract

Integrating large intelligent reflecting surfaces (IRS) into a millimeter-wave (mmWave) massive multi-input-multi-output (MIMO) technique has been a promising approach to enhance the performance of the wireless communication system with the channel state information (CSI). Most existing work assume that ideal channel estimation can be obtained, but the proposed high-dimensional cascaded MIMO channels and passive reflectors pose a great challenge to these methods. To address the abovementioned problems, we proposed a new method for the reduction of training overhead in IRS with a partial ON/OFF model and an optimizing strategy for pilot design approach. The energy consumption of large-scale antenna arrays and the pilot overhead in the training phase of signal transmission are greatly reduced. Besides, we proposed an improved deep residual shrinkage denoising network, which possesses better denoising performance with a soft thresholding model. The channel data can be denoised by deep learning methods, which greatly improve the accuracy of channel estimation. Simulation results demonstrate that the superiority of the proposed network over prior solutions.

## 1. Introduction

With the high-speed development of wireless communication systems, wave MIMO has been deployed around the high-speed railway [1–3], which can sharply improve spectral and energy efficiencies [4]. However, training overhead and hardware complexity would be significantly increased due to the use of a large number of antennas that are deployed at the base station (BS); it will cost a lot of resources to process data and is very expensive to implement on account of hardware complexity [5, 6]. In order to solve these problems, an intelligent reflecting surface (IRS) is presented as a promising technology to enhance connecting quality and reduce the training and processing consumption [7]. Global wireless data traffic has grown dramatically in the last few years. Accordingly, sixth-generation (6G) wireless communication networks are being developed to accommodate the substantial growth in mobile data rates. The IRS transmission technique is to date considered a promising technology to meet the huge requirements for high data rates in the future 6G networks [8, 9]. Specially, IRS consists of a large number of arraying reconfigurable elements which are passive and low-cost and can change the phase shifts of received signals [10].

In order to realize the advantages of low cost and reduced energy consumption in IRS-aided MIMO systems, it is essential to know the integra channel state information (CSI) [11–13]. Therefore, we denote that proceeding accurate channel estimation with reduced expense in mmWave MIMO systems is of great help to improve system performance confronting with dire challenges. Due to the reflecting elements in the IRS being passive and unable to perform signal processing [14], it is difficult to estimate the BS-IRS channel and the IRS-US channel, and this causes serious trouble in obtaining accurate channel state information. Previous channel estimation methods based on a design of a reflecting matrix by perfect CSI have been proposed in References [11–14] but still face a lot of difficulties. In Reference [11], it is pointed out that the reflection matrix can be designed with perfect channel state information to complete the channel estimation. In Reference [12], the article proposes a kind of hybrid precoding design for IRS-aided mmWave communication systems to acquire perfect CSI. In Reference [13], an intelligent reflector-enhanced wireless network by joint active and passive beamforming is proposed. Due to the high complexity of the abovementioned methods and the difficulty in obtaining perfect channel state information in the actual wireless communication environment, they are not suitable for the actual high-speed scenarios. In addition, a channel estimation method based on compressive sensing (CS) was proposed in Reference [14] with the sparsity of cascaded channels, which reduces much of the training overhead. Subsequently, in Reference [15], the authors proposed a least squares (LS) method that is based on channel estimation with switching on the reflecting elements one by one to reduce calculation complexity and resource consumption. However, the methods in References [14, 15] are applied in the frequency-flat systems with narrow-band channels. Although the estimation method in Reference [14] uses compressed sensing technology to reduce the complexity of channel estimation, the complexity of channel estimation is still high due to the existence of IRS elements. Although the proposed switching mode in Reference [15] improves the accuracy of channel estimation, it is not applicable in the case of a large number of reflection elements.

To better acquire the reflecting property of the IRS, the authors in Reference [16] deployed IRS elements in orthogonal frequency division multiplexing (OFDM) systems and applied the LS to estimate cascaded channels with one antenna in all BS and USs. In this paper, the IRS is introduced into OFDM and its communication parameters are analyzed by a simulation experiment, which resolved IRS deployment issues in a frequency-flat system with broadband channels. Nevertheless, in the massive MIMO communication system, the cascaded MIMO channels between them can be extremely high-dimensional because of the large number of antennas, so that the pilot training expense and channel dimension will become especially huge. The authors in Reference [17] proposed a channel estimation scheme based on deep learning (DL) and CS with a deep denoising network-aided CS. However, the denoising network model based on deep learning proposed in this literature has the problem of insufficient noise extraction and it takes a huge amount of time to process when the training sample size is large. In References [18, 19], two channel estimation schemes, respectively, based on compressive sensing and deep learning (DL) were proposed, whereby the angular domain channel sparsity was utilized for reduced pilot overhead, the problems of the high cost of pilot training, and high complexity of channel estimation are solved. However, the accuracy of channel estimation is low because noise is not considered.

It is clear from the abovementioned literature that the IRS-enhanced massive MIMO system has a huge overhead problem in pilot training, and it is difficult to accurately obtain channel state information through traditional channel estimation techniques due to the influence of noise in the wireless channel environment. The major contribution of this paper is to propose a reduction of training overhead based on grouping elements in the IRS with partial ON/OFF and an optimizing strategy for pilot design approach which reduces the training overhead in the communication system. The pilot optimization algorithm consists of deducing the best pilot sequence of the first antenna and then applying the shift mechanism (SM) [20] to calculate the pilot matrix of the other antennas. On this basis, we propose an improved deep residual shrinkage denoising network to further enhance the accuracy of channel estimation which possesses better denoising performance with a soft thresholding model [21].

The remainder of this paper is organized as follows.  Section 2 presents the system model and basic knowledge of handover in the IRS-aided mmWave massive communication system.  Section 3 introduces the grouping strategy of IRS elements with a partial ON/OFF model, pattern optimization of pilots based on CS, and an improved deep residual shrinkage network. Simulation and performance analysis are presented in Section 4. Finally, the paper is concluded in Section 5.

## 2. System Model

**Figure 1:**
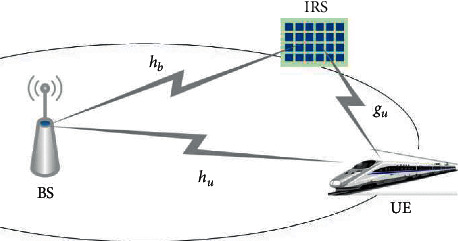
A single-user OFDM model in the IRS-aided MIMO system.

As shown in Figure 1, we consider that the IRS is set to improve the property of communication between a BS and a user [22]. In the IRS-aided mmWave massive communication system, we assume that there are *N* number of IRS elements at the IRS and the BS-IRS channel for the *k*-th reflecting element can be defined as *h*_*b*_, and the BS-user channel for the BS-User direct link is defined as *h*_*u*_. Similarly, the IRS-user channel for the IRS-user is defined as *g*_*u*_.

Specially, each element of the IRS uses an independent reflection coefficient to re-scatter the received signals, which is expressed as *ϕ*=[*ϕ*_1_,…, *ϕ*_*N*_] ∈ *C*^*N*×1^, and *ϕ*_*N*_can be written as *ϕ*_*N*_=*β*_*N*_*e*^*jθ*_*N*_^, where *β*_*N*_ denotes the amplitude coefficient and *θ*_*N*_denotes the phase shift. The concatenation of BS-IRS-user channels is defined as*h*_*b*_*∗ϕ*_*N*_*∗g*_*u*_. Therefore, the composite BS-IRS-user channel for all IRS elements can denoted by *h*_*r*_, which can be represented as follows:

(1) $$\begin{array}{c} h_{r}=V\phi \text{,} \end{array}$$

where *V*=[*v*_1_,…, *v*_*N*_] and *v*_*N*_=*ϕ*_*N*_*∗g*_*u*_. Hence, the channel impulse response (CIR) in the BS-user channel, which includes the BS-user channel and the BS-IRS-user channel, can be expressed as follows:

(2) $$\begin{array}{c} \bar{h}=h_{r}+h_{u}\text{.} \end{array}$$

In this mmWave communication system, pilot signals are sent from the user, then reflected by IRS to the BS, which estimates the channels and calculates design parameters. Without loss of generality, we attract attention on uplink communication from the user to the BS in this paper. Moreover, for downlink communication, the design parameters can be computed by channel reciprocity and leveraging time division duplexing (TDD) based on the channel information get from uplink training.

## 3. Proposed Channel Estimation Technique

In this part, we propose a new improved deep residual shrinkage network (IDSRN) with grouping IRS elements which is partial ON/OFF to enhance the accuracy of channel estimation and a kind of pilot optimization method based on CS to reconstruct the channel. This method based on CS and DL can reduce the training expense and channel estimation complexity.

### 3.1. Grouping IRS Elements with the Partial ON/OFF Model

**Figure 2:**
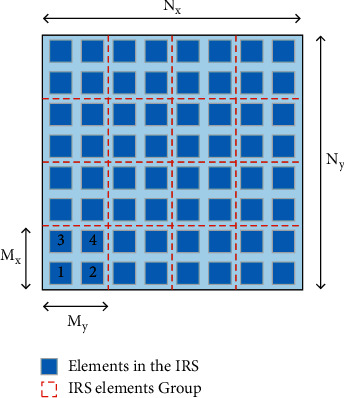
Introduction of the IRS reflect array and grouping IRS elements. *N*_*x*_ and *N*_*y*_ denote that elements in each row and column and the grouping elements, and *M*_*x*_ and *M*_*y*_ denote the elements in the group.

Because the adjacent elements are usually packed together in the uniform planar array, the channels in mmWave MIMO communication systems with IRS have a practical correlation [23]. Therefore, as shown in Figure 2, we propose a grouping design for adjacent IRS elements that form a block, in which we consider that the grouping IRS elements have a similar reflection coefficient and we switch on part of the groups instead of opening the whole elements.

The whole IRS elements' set is defined as *N* and we make *M* denote the number of groups with 1 < *M* < *N*. Therefore, we denote that the size (number of IRS elements) of each group is defined as *K*=*N*/*M*. Moreover, we define the grouping ratio as *J*, with a 1/*M* or *M*/*N*, which could be used to adjust the size of the grouping elements. For instance, as shown in Figure 2, we consider that the whole IRS elements, which has, respectively, *N*_*x*_ and *N*_*y*_ elements in each row and column, and the grouping element ratio *J* is defined as 1/4, which has *M*_*x*_ and *M*_*y*_ in each row and column. We can also change the number of groups *M* by adjusting the grouping ratio *J* and *M*_*x*_ and *M*_*y*_. Because the grouping of IRS elements has a common reflection coefficient, the IRS reflection coefficients can be re-expressed as follows:

(3) $$\begin{array}{c} \phi =\bar{\phi }⊗1_{K\times 1}\text{,} \end{array}$$

where $\bar{\phi }=\left[\bar{\phi_{1}},\ldots ,\bar{\phi_{M}}\right]\in C^{N\times 1}$ denotes that the grouping elements reflection coefficients, and $\bar{\phi_{M}}$ denotes that the common reflection coefficients in the *M*-th group. Therefore, the consolidation of the BS-IRS channel, US-IRS channel, and IRS reflection can be expressed as follows:

(4) $$\begin{array}{c} H=\left[v_{1},\ldots ,v_{N}\right]\bar{\phi }⊗1_{K\times 1}=\left[v_{1}^{\prime },\ldots ,v_{M}^{\prime }\right]\bar{\phi }=V^{\prime }\;\bar{\phi }\text{,} \end{array}$$

where *H* denotes that the reflecting channel frequency response associated with the *N*-th IRS element, and *v*_*M*_′ denotes that the channel frequency response associated with the *M*-th grouping of IRS elements.

In order to reduce the training cost and estimation complexity further, we consider that switch on the part of the grouping IRS instead of driving the whole IRS elements. Therefore, we consider that *M*-th grouping elements with their amplitude vector defined, as are switched on, and others with their amplitude vector defined as *θ*=0 represents in no reflection mode. Then, the received signal associated with *M*-th elements can be expressed as follows:

(5) $$\begin{array}{c} y_{k}=\theta XH_{k}+w\text{,} \end{array}$$

where *X* denotes the training signal, and *H*_*k*_ denotes the channel of *k*-th grouping element; *w* denotes noise in the communication environment.

### 3.2. Pattern Optimization of Pilots Based on CS

Due to the BS-IRS channels with the sparsity feature, we propose a CS-based optimization method which uses a compressed sensing technique to optimize the pattern better to solve the problem of the tremendous expense of pilot training in mmWave MIMO communication systems while enhancing the performance of signal reconstruction. This method is a kind of algorithm that can adaptively reduce the pilot vector based on the autocorrelation matrix with the shift mechanism.

We first assume that the column in the pilot matrix *X* ∈ R^*M*×*N*^ has been normalized and that the autocorrelation matrix *R* of the pilot matrix is represented as *R*=*X*^*T*^*X*. Therefore, *R* is a positive semi-defined matrix with similar diagonalization that can be defined as follows:

(6) $$\begin{array}{c} R=Q\left[\begin{array}{ccccc} \lambda_{1} & \; & \; & \; & \;\\ \; & \lambda_{1} & \; & \; & \;\\ \; & \; & \ddots & \; & \;\\ \; & \; & \; & \lambda_{M} & \;\\ \; & \; & \; & \; & 0 \end{array}\right]Q^{T}\text{,} \end{array}$$

where *λ*_1_, *λ*_2_, ⋯, *λ*_*M*_, 0 denotes that the *M* eigenvalue of matrix *R* is greater than zero, and because matrix *R* is a real symmetric matrix, it denotes that *R*=*R*^*T*^, and *Q* is an *N*-order orthogonal matrix. On account of that all diagonal elements in matrix *R* are one, the sum of the eigenvalues squared can be represented as follows:

(7) $$\begin{array}{c} \lambda_{1}^{2}+\lambda_{2}^{2}+\cdots +\lambda_{M}^{2}=trace\left(R^{2}\right)=trace\left(RR^{T}\right)={\left\|R\right\|}_{F}^{2}=N+\underset{i,j=1,2,,,N\;an\;d\;i\neq j}{\sum }{\left|r_{ij}\right|}^{2}\text{.} \end{array}$$

Then, we assume that function *f*() on the basic of the Lagrangian multiplier method which is defined as follows:

(8) $$\begin{array}{c} f\left(\lambda_{1},\lambda_{2},\cdots ,\lambda_{M},\beta \right)=\lambda_{1}^{2}+\lambda_{2}^{2}+\cdots +\lambda_{M}^{2}+\beta \left(N-\lambda_{1}-\lambda_{2}-\cdots -\lambda_{M}\right)\text{.} \end{array}$$

The partial derivative of *λ*_1_, *λ*_2_, ⋯, *λ*_*M*_can be acquired by calculation as follows:

(9) $$\begin{array}{c} \left\{\begin{array}{c} 2\lambda_{1}-\beta =0,\lambda_{1}>0\;\\ 2\lambda_{2}-\beta =0,\lambda_{2}>0\\ \ldots \\ 2\lambda_{M}-\beta =0,\lambda_{M}>0 \end{array}\right.\text{.} \end{array}$$

It denotes that a pole can be acquired in *λ*_1_=*λ*_2_=⋯=*λ*_*M*_, and in the first situation, when *λ*_1_=*N*, *λ*_2_=…=*λ*_*M*_=0, *λ*_1_^2^+*λ*_2_^2^+⋯+*λ*_*M*_^2^=*N*^2^. In the other situation, when *λ*_1_=*λ*_2_=⋯=*λ*_*M*_=*N*/*M*, *λ*_1_^2^+*λ*_2_^2^+⋯+*λ*_*M*_^2^=*N*^2^/*M* < *N*^2^.Therefore, *λ*_1_=*λ*_2_=⋯=*λ*_*M*_ is the only pole and not the maximum point, and it is the global minimum point. We can deduce (10) from the above formulas:

(10) $$\begin{array}{c} \underset{i,j=1,2,,,Nan\;di\neq j}{\sum }{\left|r_{ij}\right|}^{2}\geq \frac{N^{2}-NM}{M}=b_{d}\text{,} \end{array}$$

where *b*_*d*_ represents that the optimal lower bound which the sum of each row's autocorrelation in the pilot matrix could reach, and we average the optimal lower bound of autocorrelation to every element defined as follows:

(11) $$\begin{array}{c} p_{d}=\sqrt{\frac{b_{d}}{N^{2}-N}}=\sqrt{\frac{N-M}{M\left(N-1\right)}}\text{.} \end{array}$$

Moreover, the average column correlation of the matrix to be optimized is defined as follows:

(12) $$\begin{array}{c} P_{t}=\sqrt{\frac{\sum_{i,j=1,2,,,Nan\;di\neq j}{\left|r_{ij}\right|}^{2}}{N^{2}-N}}\text{.} \end{array}$$

To achieve the goal of reducing pilot matrix *X* column correlation, we consider that set a reduced autocorrelation matrix parameter as*P*_*s*_, and make *P*_*d*_ ≤ *P*_*s*_ < *P*_*t*_ as the form of reduction to curtail elements in the autocorrelation matrix R. The rule of reduction is defined as follows:

(13) $$\begin{array}{c} \left\{\begin{array}{c} r_{ij}=p_{s}\times sign\left(r_{ij}\right),\left|r_{ij}\right|>P_{s}\\ r_{ij}=r_{ij},\left|r_{ij}\right|<P_{s} \end{array}\right.\text{.} \end{array}$$

Usually the reduced autocorrelation matrix becomes a nonsingular matrix, but *R*=*X*^*T*^*X* limits that the pilot matrix is singular, we need to restore the nonsingular polit matrix. We apply cropping to small eigenvalues to reserve the values in the original matrix.

We can deduce formula (14) by *R*=*X*^*T*^*X*=*Q*Λ*Q*^*T*^=*Q*Λ^1/2^(*Q*Λ^1/2^)^*T*^:

(14) $$\begin{array}{c} X={\left(Q\Lambda^{1/2}\right)}^{T}\text{.} \end{array}$$

We can restore the pilot matrix by reserving the *M* maximal values in Λ matrix. Then, we assume the pilot matrix in the first antenna is matrix *X* and use the shift mechanism to calculate the pilot matrix of other antennas, which defines that if *X*={*y*_1_, *y*_2_, ⋯, *y*_*M*_} is the best pilot matrix of the first antenna and the pilot matrix of other antennas is the shift mechanism as*X*_*i*_={*y*_1_+*i*_*i*_ − 1, *y*_2_+*i*_*i*_ − 1, ⋯, *y*_*m*_+*i*_*i*_ − 1}, each *X*_*i*_ has common autocorrelation. The last output pilot matrix is {*X*_*i*_}_*i*=1_^*C*^, where C is the total number of transmitting antennas.

At least we apply simultaneous orthogonal matching pursuit (SOMP) [24] to estimating the channels with the grouping partial ON/OFF and we can acquire the estimation channel *H*=[*H*_1_, *H*_2_, ⋯, *H*_*K*_].

### 3.3. Improved Deep Residual Shrinkage Network

Because of the property that elements of the channel matrix in mmWave MIMO communication which possess high correlation, we denote that the channel matrix can be reconstructed as a two-dimensional noisy image with double channels. Therefore, we can apply the improved deep residual shrinkage network to improve the estimation accuracy. The estimation channel matrix can be represented as follows:

(15) $$\begin{array}{c} H=\tilde{H}+n\text{,} \end{array}$$

where *H*is the estimation channel matrix, $\tilde{H\;}$is the true estimation channel, and *n* is the noisy matrix.

In order to input *H*into the denoising network, we should extract the real-valued matrix and the imaginary value matrix from these estimation channel matrixes *H* ∈ *ℂ*^*N*^ separately which can be defined as follows:

(16) $$\begin{array}{c} O=\left[I\left(H\right),R\left(H\right)\right]\in R^{N\times 2}\text{,} \end{array}$$

where *I*(*H*) denotes the imaginary value matrix and *R*(*H*) denotes the imaginary value matrix.

**Figure 3:**
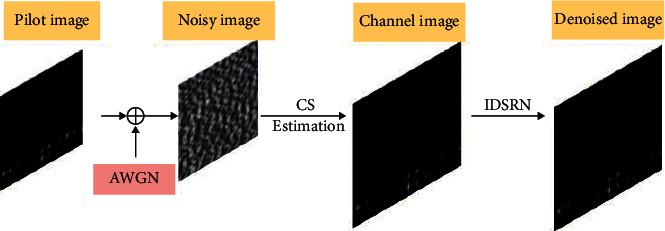
(a) The process of denoising channel image. We apply the channel estimation method based CS to acquiring channel image, and AWGN represents noisy environment, and IDRSN is the proposed network.

Then, we can reconstruct the channel matrix *O*into a two-dimensional noisy image with double channels as the input of this network, which will be introduced in this section.  Figure 3 shows the process of the denoising channel image.

#### 3.3.1. The Architecture of the Network

**Figure 4:**
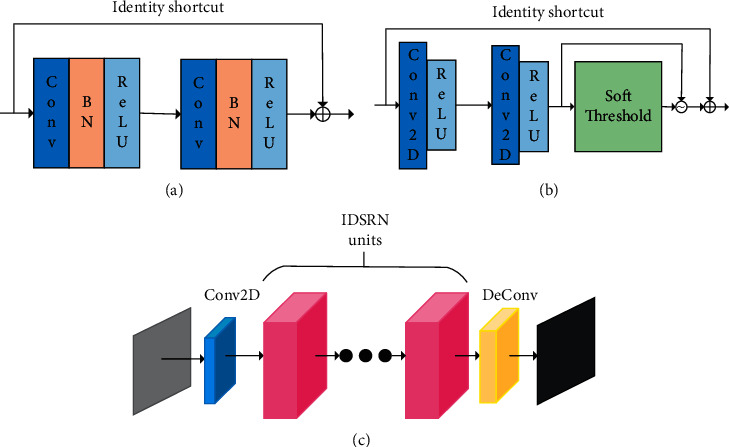
(a) Building unit entitled ResNet. (b) Building unit entitled IDSRN units. (c) Architecture of IDSRN.

The improved deep residual shrinkage network (IDRSN) is a new multiscale method based on the common deep residual shrinkage network (DRSN). Recently, residual networks [25](ResNet) have attracted much attention of people in the field of deep learning. As shown in Figure 3, the residual basic unit (RBU) consists of batch normalization (BN) layers, two rectified linear units (ReLU), two convolutional layers, and an identity shortcut which is the most important component of ResNet. However, in the IDRSN, the basic component of which is shown in Figure 4 and which consists of two ReLUs, two convolutional 2D layers [26], a shortcut, and a soft thresholding model. As shown in Figure 4, the whole architecture of IDSRN consists of an input layer, a convolutional 2D layer, ten numbers of IDRSN-RBUs, a deconvolution layer, and an output layer.

In (c), Conv denotes the complicated convolutional layer, and DeCon denotes the deconvolutional layer which is used to reconstruct the channel image.

In the improved deep residual shrinkage network, we apply a complicated convolutional layer named the convolutional 2D layer instead of a conventional layer to better processing the data in the complex domain, which is expressed as follows:

(17) $$\begin{array}{c} \left[\begin{array}{c} R\left(W*h\right)\\ I\left(W*h\right) \end{array}\right]=\left[\begin{array}{cc} A & -B\\ B & A \end{array}\right]*\left[\begin{array}{c} x\\ y \end{array}\right]\text{,} \end{array}$$

where *W*=*A*+*Br·* denotes the complex filter matrix and *h*=*x*+*ry* denotes a complex vector as the input of the convolutional layer.

#### 3.3.2. IDSRN Units

**Figure 5:**
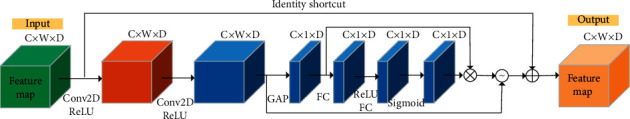
In the IDSRN units, (C) W, D in *C*×*W*×D denote the number of channels, width, and height of the feature map.

On account of that the BN layer has terrible influence in the network of picture processing which can break the correlation of the signal, we consider to apply DSRN units without BN layers to construct IDSRN units and replace all common convolutional layers with multiscale convolutional layers so that improving the property of extracting feature.  Figure 5 shows that the IDSRN units, which use the soft thresholding to remove noise in features maps and which as a nonlinear transformation layer into the network units.

**Figure 6:**
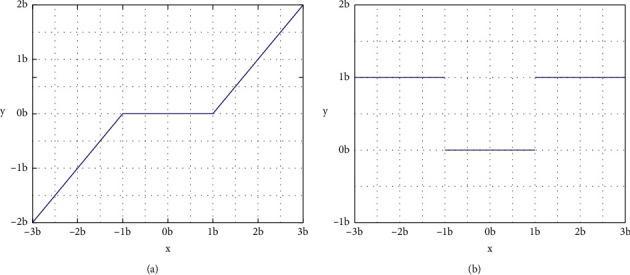
Illustration of (a) soft thresholding and (b) its derivative.

The function of soft thresholding in Figure 6 is expressed as follows:

(18) $$\begin{array}{c} Y^{\prime }=\left\{\begin{array}{c} Y-b,Y>b\\ 0,-b\leq Y\leq b\\ Y+b,Y<-b \end{array}\right.\text{,} \end{array}$$

where *Y*′is the output feature map, *Y*is the input feature map, and *b*is the threshold. Moreover, we can consider that the derivation of the output on input between processing of the soft thresholding is either one or zero, which can be represented as follows:

(19) $$\begin{array}{c} \frac{\partial Y^{\prime }}{\partial x}=\left\{\begin{array}{c} 1,Y>b\\ 0,-b\leq Y\leq b\\ 1,Y<-b\; \end{array}\right., \end{array}$$

which means that the threshold can keep off the gradient vanishing and exploding problems that can be acquired by the soft thresholding module.

In this module, the feature map as the input of this module passed the global average pooling (GAP) to get a one-dimension vector. Then, a two-fully connected (FC) layer network with a sigmoid function is applied to the one dimension's vector to acquire a scaling parameter which is scaled to the range of (0,1). The parameters can be expressed as follows:

(20) $$\begin{array}{c} \alpha =\frac{1}{1+e^{-t}}\text{,} \end{array}$$

where *t* is the output of the soft thresholding module, and*α* denotes the corresponding scaling parameter. The threshold can be acquired by the scaling parameter multiplying the average value of |*t*|. After the soft thresholding module, we input *t* to the deconvolution layer to get the original size noiseless image.

In the IDSRN, each ReLU can be expressed mathematically as follows:

(21) $$\begin{array}{c} y=\max\;\left(x,0\right)\text{,} \end{array}$$

where *x* and *y* are the input and output of the activation function, respectively, and it accelerates the training process and solves the problem of gradient disappeared.

#### 3.33. Training Network

In the improved deep residual shrinkage network, we consider adopting the mean square error (MSE) as the loss function of this network, which is be defined as follows:

(22) $$\begin{array}{c} L\left(\xi \right)=\frac{1}{N}\overset{N}{\underset{i=1}{\sum }}{\left\|\rho \left({\tilde{H}}^{i};\xi \right)-\left({\tilde{H}}^{i}-H^{i}\right)\right\|}^{2}\text{,} \end{array}$$

where *N* denotes the total number of samples and *i* denotes the data index, and *ξ* denotes the parameters in IDSRN, and *ρ* denotes a residual mapping for noise, such as $\rho \left({\tilde{H}}^{i}\right)\approx n$ which should be learned in a deep learning network. We consider to apply the simulated channel dataset generated by the classical channel model [27] to avoid the contingency of training samples and train the IDSRN offline. The trained network in this paper can learn the mapping from$\tilde{H}$ to the channel noise *n*^*e*^as $\rho \left(\tilde{H}\right)\approx n$ and the enhanced channel estimation can be represented as follows:

(23) $$\begin{array}{c} H^{e}=\tilde{H}-\rho \left(\tilde{H}\right)=H+n-n^{e}\text{.} \end{array}$$

The IDSRN uses the Adam optimizer to optimize weight in the network, and the batch size is set as 16 with 800 epochs. We consider to feed the network with 4,000 training samples in the training process, and the initial learning rate is set as 0.01 and descends to 0.8 times of the last epoch with patience 20.

## 4. Simulation and Results

We consider to adopt the normalized MSE as an evaluation index of denoising property in IDSRN, which can be expressed as follows:

(24) $$\begin{array}{c} NMSE=\frac{1}{N}\overset{N}{\underset{i=1}{\sum }}\frac{{\left\|H-H^{e}\right\|}_{F}^{2}}{{\left\|H\right\|}_{F}^{2}}\text{.} \end{array}$$

In our simulation, we consider the mmWave MIMO system carrying out at 28 GHz with the bandwidth is *f*=100*MHz* and the number of OFDM's subcarriers *K*=256 in the phase of training pilot. The number of IRS elements are set as 64 and the cyclic prefix (CP) is set to:*L*_*CP*_=32. We consider that *L*=6 and azimuth/elevation AoAs and AoDs are set as uniform distribution *u*(−*π*/2, *π*/2).

The deep learning-related settings used in this paper are shown in Table 1.

**Figure 7:**
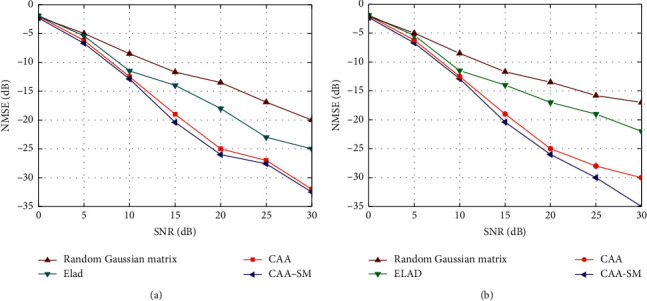
MSE performance of CAA, Elad algorithm, random Gaussian matrix algorithm, and CAA-SM in the communication system with different number of antennas. (a) Process of four antennas and (b) Process of eight antennas.

First of all, in order to investigate the feasibility of pilot training reduction, we applied different pilot optimization methods in communication systems with 4 antennas or 8 antennas and acquired the NMSE of preliminary estimation by the SOMP algorithm (*β* = 4). In order to prove the effectiveness of pilot optimization algorithm based on SM and compressed sensing technology, we named SM-enhanced corresponding adaptive autocorrelation (CAA-SM) pilot matrix optimization algorithm in the second part of this paper. The proposed method and pilot optimization reconstruction algorithms based on random Gaussian matrix, the Elad method, and the corresponding adaptive autocorrelation (CAA) method are, respectively, used for channel estimation using the SOMP estimation algorithm. As shown in Figure 7, it can be observed that as the SNR increases, CAA-SM performs nearly with CAA in the communication system processing of 4 antennas and acquires better performance than others. Meanwhile, the CAA-SM also achieves better performance in the communication system processing of 8 antennas. In the case of multiple antennas with high SNR, the CAA-SM achieves a performance gain of around 3 dB with CAA, indicating that which can obtain better channel estimation performance under the condition of multiple antennas with high SNR. This is because with the enhancement of SM, the computational complexity is greatly reduced, and the efficiency and accuracy of channel estimation are greatly improved. In either case, the estimation error of the Elad algorithm and the random Gaussian matrix method is large. The random Gaussian matrix method has a large variance of column correlation, which is not conducive to channel reconstruction and estimation. The setting of reduction parameters of the Elad method will seriously affect the performance of pilot optimization, which has great limitations. We consider to choose CAA-SM to optimize the pilot matrix with training reduction and time consumption for the following simulations.

In order to analyze the influence of the packet strategy of the IRS reflection element on channel estimation and the feasibility of the SOMP estimation algorithm, simulation experiments are carried out under the conditions that the number of reflecting elements is 10×10 and the packet strategy is 1, 2, and 4.

The packet policy parameters are shown in Table 2.

**Figure 8:**
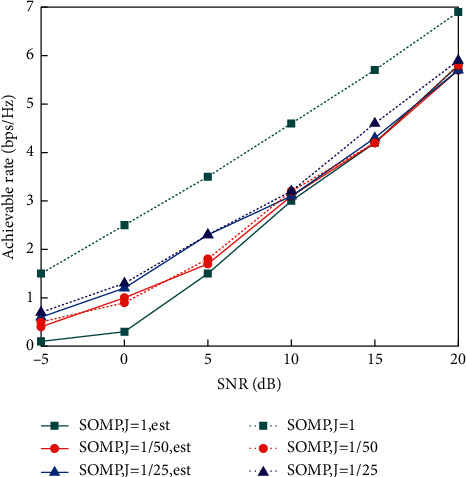
Achievable rates of different packet strategy of IRS reflection element with SOMP algorithm.

In this experimental analysis, the channel achievable rate is used as the evaluation index, and the number of measurements of the SOMP estimation algorithm is 100. The simulation results are shown as follows:

As can be seen from Figure 8, with the increase in SNR, the achievable rates of the three different packet strategies gradually increased. When the SNR is low, the channel achievable rate under the *J* = 1 packet strategy is the lowest, and it is more sensitive to the error in channel estimation. The channel achievable rate under the *J* = 1/25 packet strategy is the highest. There is little difference in channel estimation performance between the three packet strategies. It can also be seen from Figure 8 that, regardless of high or low SNR, when packet strategy *J* = 1, the gap between the channel estimated achievable rate and that under perfect channel state information is large, while when packet strategy *J* = 1/25 and *J* = 1/50, the gap between the channel estimated achievable rate and that under perfect channel state information is small and the estimation performance is good. This is because compared with the IRS reflection elements that need to be turned on one by one before any grouping, the packet strategy can be used to turn on more reflection elements at a time, which can receive higher SNR and have better channel estimation performance. In the subsequent simulation experiment analysis, the packet strategy with *J* = 1/25 will be used for experimental simulation.

**Figure 9:**
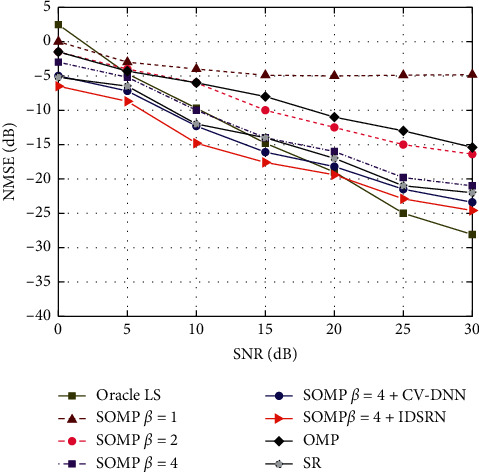
Robustness of the proposed IDSRN network and pretrained with SNR = 10 dB and (L) = 6 is robust to various SNRs.

The proposed network is simulated and analyzed to prove the robustness and enhancement of accuracy for preliminary estimation, as shown in Figure 9.

The channel estimation algorithm based on the IDSRN model proposed in this paper is compared with the channel estimation algorithm with different sampling rates without past noise processing, the channel estimation algorithm based on deep learning, and Oracle-LS. As shown in Figure 9, our proposed algorithm shows a performance gain of around 5 dB with CV-DNN accounting of which has a powerful denoising ability and extraction performance inherent characteristics, acquired better estimation property than other schemes. Compared with the OMP algorithm, the proposed algorithm has higher accuracy, because the SOMP algorithm synchronizes data and has higher accuracy compared with the OMP algorithm. In the meanwhile, compared with the channel estimation algorithm based on deep learning, which is named the SR algorithm, the proposed algorithm takes into account the bad influence of noise factors in the wireless channel on the channel estimation and performs denoising processing on the channel data, resulting in better estimation performance.

## 5. Conclusions

In this paper, in order to reduce the consumption of pilot training, we propose a CA-SSM pilot optimization method based on CS to reconstruct the channel and optimize the pilot matrix. Due to prior estimation algorithms' existing problem of poorer denoising property and estimation performance, we propose an IDSRN network with Grouping IRS Elements which is partial ON/OFF to enhance the accuracy of channel estimation, which can reduce channel estimation complexity. Meanwhile, the proposed network in this work comprises of better extraction performance for channel characteristics, which contributes to the enhancement of a system property. Moreover, through the simulation with the proposed pilot optimization method and IDSRN model, we demonstrate that IDSRN has enough robustness, which makes the model can be used in different SNR scenarios by offline training.

Although the experimental analysis part of proposed method is lack of data validation of the real scene, with the continuous development of the IRS technology and application, in the subsequent experiments in this article will introduce the application of the IRS after experimental data and the algorithm was demonstrated. At the same time, in the future research, we will conduct channel estimation and modeling analysis for the communication system with IRS elements deployed in different terrains, such as hilly scene and plain scene.

## Figures and Tables

**Table 1:** Parameter settings of the experimental test.

Name	Specific parameters
CPU	i5-7300HQ CPU @ 2.50 GHz
Hard disk	1 TB SSD
Graphics card	NVIDIA GeForce RTX 2060 SUPER
Memory	8G
Operating system	Windows 10
Framework	IDRSN
Accelerated environment	CUDA 11.5
TensorFlow	1.0.1
Keras	2.2.1

**Table 2:** Group parameter setting of IRS elements.

J	1	1/50	1/25
K	100	2	4
*M*_x_×*M*_y_	1×1	5×10	5×5

## Data Availability

The data used to support the findings of this study are available from the corresponding author upon request.

## Abbreviations

BS: Base station
IRS: Intelligent reflecting surface
MIMO: Multi-input-multi-output
6G: Sixth-generation
CSI: Channel state information
CS: Compressive sensing
LS: Least squares
OFDM: Orthogonal frequency division multiplex
DL: Deep learning
SM: Shift mechanism
TDD: Time division duplexing
IDSRN: Improved deep residual shrinkage network
SOMP: Simultaneous orthogonal matching pursuit
DRSN: Deep residual shrinkage network
RBU: Residual basic unit
BN: Batch normalization
ReLU: Rectified linear unit
MSE: Mean square error
CP: Cyclic prefix
CAA: Correlation of adaptive auto.
